# Improving Fine Control of Grasping Force during Hand–Object Interactions for a Soft Synergy-Inspired Myoelectric Prosthetic Hand

**DOI:** 10.3389/fnbot.2017.00071

**Published:** 2018-01-10

**Authors:** Qiushi Fu, Marco Santello

**Affiliations:** ^1^Neural Control of Movement Laboratory, School of Biological and Health Systems Engineering, Arizona State University, Tempe, AZ, United States; ^2^Mechanical and Aerospace Engineering, University of Central Florida, Orlando, FL, United States

**Keywords:** neuroprosthetics, hand function assessment, object manipulation, grasping, haptic feedback, force control

## Abstract

The concept of postural synergies of the human hand has been shown to potentially reduce complexity in the neuromuscular control of grasping. By merging this concept with soft robotics approaches, a multi degrees of freedom soft-synergy prosthetic hand [SoftHand-Pro (SHP)] was created. The mechanical innovation of the SHP enables adaptive and robust functional grasps with simple and intuitive myoelectric control from only two surface electromyogram (sEMG) channels. However, the current myoelectric controller has very limited capability for fine control of grasp forces. We addressed this challenge by designing a hybrid-gain myoelectric controller that switches control gains based on the sensorimotor state of the SHP. This controller was tested against a conventional single-gain (SG) controller, as well as against native hand in able-bodied subjects. We used the following tasks to evaluate the performance of grasp force control: (1) pick and place objects with different size, weight, and fragility levels using power or precision grasp and (2) squeezing objects with different stiffness. Sensory feedback of the grasp forces was provided to the user through a non-invasive, mechanotactile haptic feedback device mounted on the upper arm. We demonstrated that the novel hybrid controller enabled superior task completion speed and fine force control over SG controller in object pick-and-place tasks. We also found that the performance of the hybrid controller qualitatively agrees with the performance of native human hands.

## Introduction

Restoring hand function through prostheses in individuals with upper limb loss is critically important to help them regain independence and improve quality of life. Unfortunately, the current state of commercially available prosthetic hands is still far from achieving human level dexterity, even in relatively simple object grasping tasks. Limitations in the reliability, function, and robustness of hand prosthesis has led to little use or abandonment of advanced terminal devices, as these factors are considered to be most important to the amputees (Atkins, 1989; Atkins et al., 1996; Biddiss and Chau, 2007a,b). Human-inspired approaches have been recently proposed to tackle this challenge through novel mechanical design (Godfrey et al., 2013), intuitive control (Ajoudani et al., 2014; Jiang et al., 2014), and sensory feedback (Clemente et al., 2015). Specifically, by observing how the human neuromuscular system solves the sensorimotor complexity of controlling hand movements during grasping tasks, it was found that hand postures used to grasp a large set of common objects can be approximated by a few finger joint coordination patterns, i.e., synergies (Santello et al., 1998, 2002). This implies a synergy control scheme in which controlling a large number of degrees of freedom could be simplified by using a reduced set of neural signals [for review, see Santello et al. (2013, 2016)]. By combining the concept of synergy with soft robotics technologies, a prosthetic hand, the SoftHand-Pro (SHP), was developed to simultaneously maximize simplicity and functionality (Godfrey et al., 2013). Specifically, this hand employs an under-actuated design where the number of synergies, and thus, the number of actuators, was reduced to one, i.e., the first principal component observed in human grasping data that accounts for more than 50% of the variance in grasp posture data (Santello et al., 1998). Movement from the motor was transmitted to all 19 finger joints of the SHP by means of a single tendon, hence the SHP follows the movement described by the first synergy for human grasping: flexion and adduction of the metacarpal-phalangeal and inter-phalangeal joints of the fingers, accompanied by flexion and palmar abduction of the thumb. This design is combined with an elastic recoil force implemented as elastic ligaments in all joints to help the fingers conform to arbitrary object shapes, and bring the fingers back to their starting position. These ligaments also accommodated temporary joint displacements during unexpected perturbations through hyperextension and/or torsion. Such flexibility avoids stress that could damage the hand and the environment, while enabling versatility to grasp a wide variety of objects. The embedded flexibility in the mechanical design also simplifies myoelectric control with surface electromyography (sEMG), as the user does not need to generate a sequence of muscle activation to produce hand postures that match different object shapes. Indeed, only two sEMG channels from a pair of antagonistic muscles are needed to operate the hand efficiently in individuals with upper limb loss (Godfrey et al., 2017). Although the SHP demonstrated human-like motion during reach-to-grasp (Fani et al., 2016), its capability to interact with objects with human-like force remains to be systematically validated. Such human-like force control is important in activities of daily living (ADL), which includes but are not limited to moving delicate objects, modulating grasp force to object weight, and manipulate compliant objects.

The main objective of the current study was to improve force control of the SHP. The default control gain of the SHP is tuned to enable fast free motion response, but the motor current (and, therefore, grasp force) ramps up quickly after the SHP contacts the object. This makes it difficult for the user to modulate the grasp force to the desired level (Gailey et al., 2017). In fact, a recent study, individuals with upper limb loss using SHP did not exhibit proper modulation of grasp force when lifting objects with different weights, even with the help of a mechanotactile haptic feedback device (Godfrey et al., 2016). One way to solve this limitation is to let the user modulate the control gain through co-contraction of the muscles (Ajoudani et al., 2014), such that a high co-contraction level can be mapped to high stiffness of the SHP. However, this approach could increase the complexity of myoelectric control, as the user would have to adjust co-contraction level while exerting differential activity between the flexor and extensor. Another approach is to use a force-position hybrid control scheme to handle motion and force automatically within the hand based on feedback from force/position sensing (Engeberg et al., 2008; Engeberg and Meek, 2013). However, such controller relies on accurate measurement of finger force and position in a prosthetic hand with rigid structure, and therefore, it is not fully compatible with SHP, a device designed to be mechanically compliant with only synergistic sensing of force and position across all fingers. Therefore, we propose a novel approach that automatically switches control gain based on grasping context detected from combined information from force, position, and EMG channels. This approach will be tested against the conventional SHP controller, as well as human hands, in functional tasks that require fine control of grasp forces.

## Materials and Methods

### Subjects

Sixteen subjects enrolled in the study (nine females and seven males, ages 19–34 years). They had normal or corrected-to-normal vision, and no history of musculoskeletal or neurological disorders. All subjects were naive to the experimental purpose of the study and gave informed consent to participate in the experiment. The experimental protocols were approved by the Institutional Review Board at Arizona State University in accordance with the Declaration of Helsinki. Before data collection, subjects signed an informed consent and completed the Edinburgh Handedness Questionnaire. Fifteen subjects were right-handed, and one subject was ambidextrous. They were randomly assigned to two “controller” groups [i.e., single-gain (SG) and hybrid-gain (HG) controllers, see below].

### Experimental Apparatus

**Figure 1:**
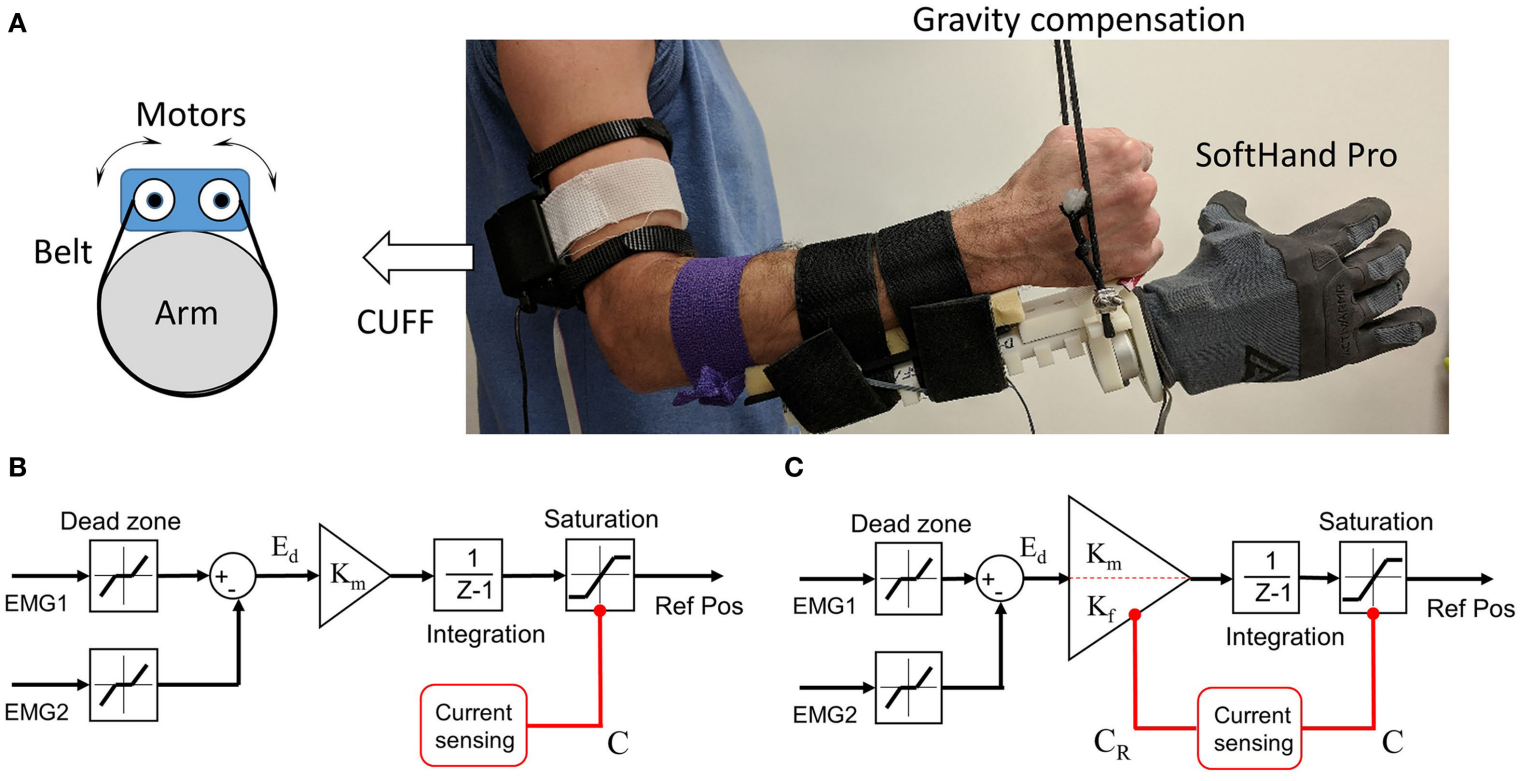
Prosthetic system implementation. **(A)** Complete setup with integration of SoftHand-Pro, Clenching Upper-limb Force Feedback device (CUFF), and gravity compensation. **(B)** Design of the Single-gain myoelectric controller. **(C)** Design of the hybrid-gain myoelectric controller.

For the present investigation of myoelectric controllers for hand prostheses, we used the SHP which is a soft robotic hand inspired from human hand synergies (Godfrey et al., 2013). Although we tested only able-bodied subjects, it has been shown that transradial amputees are able to use SHP effectively in ADL (Godfrey et al., 2017). In addition to the SHP, each subject wore a *C*lenching *U*pper-limb *F*orce *F*eedback device (CUFF) for haptic feedback of the grasping force (Casini et al., 2015). Finally, we built a gravity compensation system to off-load the weight of harness worn by subjects on their forearm and the SHP, thus minimizing fatigue (Figure 1A). We describe these systems below.

#### SoftHand-Pro

The SHP is the prosthetic version of the Pisa/IIT SoftHand (Catalano et al., 2014). The size and weight of the SHP were designed to approximate a large male hand. The electronic control board was enclosed in the back of the hand. A glove is used to cover the joints and increase contract area and friction. The battery was placed on user’s body and connected to the hand through a cable. For testing with able-bodied subjects, a customized socket interface was used to mount the SHP on their forearms (Figure 1A). Importantly, as part of the interface, we used a Quick Disconnect Wrist (Hosmer 61921, Fillauer LLC, TN, USA) to allow task-specific manual adjustment of supination/pronation angle. This ensures subjects to maintain a neutral supination/pronation angle with their own wrist throughout the experiment. The onboard microcontroller drives the motor with PID position/current control. It also communicates with EMG sensors and external programs. For myoelectric control, we used two sEMG electrodes that are commonly used for myoelectric prostheses (13E200 Myobock electrodes, Otto Bock, Germany). These electrodes are equipped with a logarithmic sensitivity adjustment and high common-mode rejection in the low frequency range (>100 dB at 50 Hz). The output of the electrodes was appropriately filtered and rectified. We placed the electrodes over m. flexor digitorum superficialis (FDS) and m. extensor digitorum communis (EDC) for flexion and extension, respectively. The difference between the sEMG magnitude measured from flexor and extensor muscles is used to drive the change of the reference motor position for the SHP (see below). This type of velocity-based proportional control allows users to scale the speed of the finger motion by modulating their EMG activities, as well as to minimize fatigue.

The SHP does not have force sensors, and it estimates the overall grasp force by current sensor. This approach takes advantage of the synergy design, since all fingers are connected by a single cable to one motor. Therefore, the grasp forces of all fingers can be transmitted to this cable, absorbing current from the motor. The motor total current (*C*) is the sum of grasp force-dependent and motor kinematics-dependent (*C_K_*) components. The latter component can be calibrated with a model that consists of position, velocity, and acceleration terms (Ajoudani et al., 2014; Casini et al., 2015). After proper calibration, the grasp force-dependent current (Residual Current, *C*_R_) can be estimated as the difference between *C* and *C_K_*. It has been demonstrated that relation between the overall grasp force and the residual current is approximately linear (Casini et al., 2015).

##### SG Controller

The SG controller is mostly identical to the best performing SHP motion controller demonstrated by Fani and colleagues (Fani et al., 2016). A small modification was made to dynamically limit the reference position. Specifically, this EMG-to-motion mapping uses the difference between the sEMG signals from wrist flexor and extensor muscles to drive the SHP. After a signal dead zone of 2% MVC was applied to each channel, the channel differential *E*_d_ was used to drive the change of SHP motor reference position with a predetermined gain *K*_m_ based on preliminary testing and previous studies (Figure 1B). Therefore, the sign and the magnitude of the differential *E*_d_ dictate the direction and velocity of the finger movement during free motion, respectively. Furthermore, we used an adaptive motor position limit which prevents the increase of reference position if the motor total current C is close to the max capacity. This prevents the reference position “closing into” the object too much, thus allowing consistent opening motion from objects with any size. Eight subjects were assigned to use the SG controller (SG group).

##### HG Controller

As mentioned earlier, the main drawback of the SG differential controller is that it cannot adapt to both free motion control and grasp force control equally well, if the reference position changes too quickly. To overcome this problem, we created a HG controller. The overall design of the HG controller is similar to the SG controller. However, the EMG-to-motion gain changes adaptively depending on the state of the SHP (Figure 1C). We defined three sensorimotor states of the SHP using the residual current C_R_ as well as the EMG differential E_d_. Specifically, Free Motion state is when the grasp force is 0 or very low, i.e., *C*_R_ = 0. Fine Force state is when grasp force is above minimum and the user is trying to control grasp force, i.e., *C*_R_ > 0 and *E*_d_ > 0. The last state, Quick Release, is when grasp force is above minimum and subject is trying to quickly release the grasped object i.e., *C*_R_ > 0 and *E*_d_ < 0. We used a large gain *K*_m_ for both Free Motion and Quick Release states, and a small gain *K*_f_ for Fine Force state (see Table 1). Eight subjects were assigned to use the HG controller (HG group). We would like to emphasize that the adjustable gain is used to map EMG activity to the reference position of the motor. Unlike previous work (Ajoudani et al., 2014), the control gain for the internal motor control loop remain unchanged, therefore preserving the stability during the passage from one state to another.

**Table 1:** Summary of sensorimotor states for the hybrid-gain controller.

SoftHand-Pro states	Residual current *C*_R_	EMG differential *E*_d_	Control gain
Free motion	*C*_R_ = 0	Any	*K*_m_
Fine force	*C*_R_ > 0	*E*_d_ > 0	*K*_f_
Quick release	*C*_R_ > 0	*E*_d_ < 0	*K*_m_

#### Clenching Upper-Limb Force Feedback Device

The force feedback device CUFF used in this study has been demonstrated to enable intuitive modulations of grasp forces and correct softness discrimination (Ajoudani et al., 2014; Casini et al., 2015). Briefly, the CUFF is comprised of two DC motors attached to an elastic belt worn around the upper arm (Figure 1A). When the motors spin in opposite directions to tighten or loosen the band on the arm, the pressure around the arm applied by the band would increase or decrease, respectively. This type of mechanotactile cues provides the same modality of somatosensation as the one involved in the hand–object interactions (e.g., grasping), although at a different location. This may have advantages over other types of haptic feedback due to its ability to deliver natural feeling of force/pressure (Li et al., 2017). When the subjects use CUFF and SHP as an integrated system, the SHP estimates the grasp force using residual current *C*_R_ which is then linearly mapped to CUFF motor positions. Therefore, the grasp force can be proportionally delivered as pressure through the CUFF. Specifically, due to differences in the biomechanical characteristics of arms, we calibrated *C*_R_ to CUFF motor mapping for each subject. The automated calibration procedure finds the motor position range when the CUFF motor current reaches high and low threshold. Then the full range of *C*_R_ is linearly mapped to CUFF motor position range.

#### Gravity Compensation System

In most studies of hand prosthetics with able-bodied subjects, the prosthesis is either mounted on subject’s arm or fixed on the table separately from the subject. Both approaches are suboptimal in the investigation of object manipulation. When the prosthetic hand is mounted on the arm, healthy subjects have to overcome significant added weight to move the system. This could lead to muscle fatigue in long period of testing, thus negatively impact subjects’ performance. Additionally, the weight from the hand prosthesis may influence subject’s perception of the object physical property, preventing modulation of grasping force in response to object weight. In contrast, if the prosthetic hand is detached from the subjects, the experiments could not assess the hand-arm coordination (e.g., reach to grasp), which is an important component of natural hand-object interactions (Grafton, 2010; Davare et al., 2011). To overcome these drawbacks, we built a gravity compensation system that offsets the gravitational force created by wearing the prosthetic hand (Figure 1A). This system is functionally similar to the one developed in Wilson et al. (2017). Specifically, we use a light cable and a series of pulleys to connect the wrist part of the prosthesis to a counter-weight. The counter-weight has the same weight as the entire hand prosthetics (SHP and harness) worn by the subjects. This system helped to prevent fatigue in our study, which required intensive repetition of hand movement over more than 1 h of testing.

### Experimental Protocol

Our study consisted of three sessions: (1) baseline trials: experimental tasks with normal right hand, (2) training trials: training tasks with SHP and CUFF, and (3) SHP trials: experimental tasks with SHP and CUFF. We use the data from normal hand as a benchmark to evaluate the performance of the prosthetic system. The tasks used in our study are described below.

#### Training Tasks

We developed a two-step simple training scheme that helps subjects to familiarize with myoelectric control of the SHP and haptic feedback from the CUFF.

##### Motion Control Training

**Figure 2:**
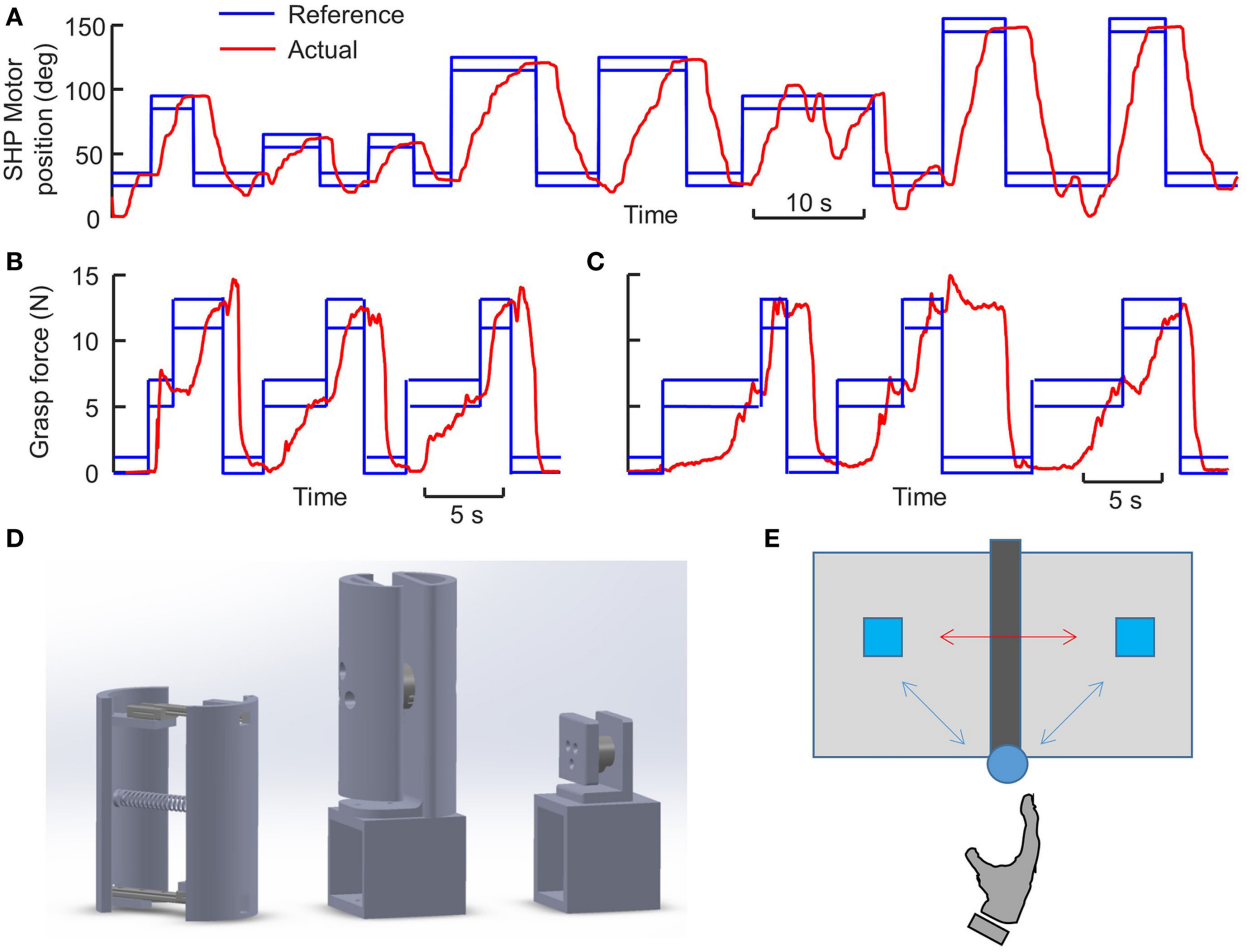
Training and experimental tasks. **(A)** Representative trial of motion control training. **(B)** Representative trial of force control training from hybrid-gain group. **(C)** Representative trial of force control training from single-gain group. **(D)** Design of the objects used for training and experimental tasks. From left to right: Compliant object, Large object, Small object. The compartments at the bottom of Large and Small objects allow additional mass to be inserted and changing the weight of the objects. Large object can be set to 820 or 420 g, whereas Small object can be set to 420 or 220 g. The force sensors in the middle of Large and Small objects measure grasp forces, which can be used to render the Fragility of the objects. **(E)** Top view of the setup for object pick-and-place task.

The objective of this training was to help subjects learn the EMG-to-motion mapping of the SHP. No CUFF feedback was given during this task. Subjects sat comfortably wearing the prosthetic system, with their forearm resting on the table. We adjusted the quick connector at the wrist to have the SHP 90° supinated, such that palm of the SHP facing upwards. A monitor was placed in front of the subjects, showing continuous visual feedback of the motor position of the SHP, as well as a target motor position. Subjects were required to control the open and close of the SHP to match the target motor positions as quickly as possible (0° and 170° are fully open and close, respectively). We defined five levels of target motor position: 30°, 60°, 90°, 120°, and 150°. The target position automatically advanced to the next if the actual motor position stays within target with an error margin of ±5° for an accumulated 1 s. There were three trials for this task. Each trial consists of eight “close and open” actions that always start from 30° and move to one of the other positions, then move back to 30° (Figure 2A).

##### Force Control Training

The objective of this training is to help subjects learn the haptic feedback given by the CUFF. We directly measure grasp force with a cylindrical object fixed to the table. The object is split into two grasp surfaces with a Force/Torque sensor (Nano 25, ATI Industrial Automation, NC, USA) is installed in the center (Figure 2D). Each grasp surface is a curved surface (3.25 cm radius 150° arch) with a height of 12 cm. In this task, the quick connector at the wrist was adjusted to neutral position to allow natural power grasp around the cylindrical object, with thumb and fingers of the SHP placed on each grasp surface. Visual feedback of the actual grasp force and target force were shown on the monitor. To get ready for each trial, subjects were instructed to move the SHP to the close proximity of the cylindrical object. Upon hearing a “Go” cue, subjects control the SHP to grasp on the object and they were instructed to match the target force as quickly as they can. Three target levels of grasp force were defined and repeated three times in the same order within a trial: 6, 12, and 0 N (Figures 2B,C). Similar to motion training, the target force automatically advanced to the next if the actual grasp force stays within target with an error margin of ±1 N for an accumulated 1 s. There was a total of five training trials. Most importantly, subjects were told that the pressure applied by the CUFF is proportional to the displayed grasp force, and they should familiarize themselves with the CUFF feedback.

#### Experimental Tasks

To assess the performance of two myoelectric controllers, we developed the following three Experimental Tasks. They were inspired by commonly used clinical hand function assessment tools (e.g., Southampton Hand Assessment Procedure, Block and Box Test, etc.), with the focus on the ability of fine control of grasp forces during functional use of the prosthetic hand. Note that these tasks were performed with native right hand and the SHP in baseline trials and SHP trials, respectively.

##### Large Object Pick and Place

Grasp and transport object is one the most common activities in daily life. Subjects were instructed to pick and place a cylindrical object (Figure 2D) with power grasp repetitively. The object was the same as the one used in the CUFF familiarization task, but it was free to move instead of being fixed to the table. Additionally, the weight of the object can be modified by inserting mass into the base of the object. There were two object weights: Medium (420 g) and Heavy (820 g). A soft mat was placed on the table in front of the subjects to prevent damage to the object if dropped. We marked two *target regions* separated by 30 cm, and a 5-cm high metal bar was placed on the mid-line between the two target regions as an obstacle (Figure 2E). Subjects had to align their right shoulder with this obstacle. The proximal end of the obstacle was defined as the *start region*, which was 30 cm away from the right shoulder. For SHP trials, we set the wrist at neutral position to enable natural grasp posture (i.e., thumb and fingers of the SHP placed on each grasp surface). Subjects were asked to start a trial with either their normal hands or the SHP in the start region, and the object started in the right target region.

On an auditory “GO” signal, subjects reached to grasp the object and transport it to the other target region and move their hand back to the start region, and repeat this process as many times as possible successfully within 45 s. A successful transport was recorded if the object was not dropped or “crushed.” The crushing of the object was rendered by giving “glass breaking” sound when the force normal to the grasp surface exceeded a pre-defined *crushing threshold*. There were two types of fragility. The Solid type had a crushing threshold of 80 N, therefore subjects did not need to be careful about crushing the object. The Fragile type had a crushing threshold defined based on the object weight, such that the threshold is ~3 N above the minimum grip force required to prevent slipping. The coefficient of friction between the glove and the object was estimated to be 0.5. Therefore, the fragile crushing threshold for the Medium and Heavy objects was 6 and 9 N, respectively. We instructed subjects to replace the object to the closest target region if the object is “crushed,” and they can retry without completely release the object. The kinematics of the object was tracked by motion capture system with a marker placed on the base of the object (Impulse, Phasespace, Inc.).

##### Small Object Pick and Place

This task was similar to the Large object pick-and-place task, the only difference being that the object is smaller (Figure 2D). Specifically, there were two small grasp surfaces (size: 3.5 cm × 4 cm, 3 cm distance) and subjects were required to use a three-digit precision grasp (tips of thumb, index, and middle finger). For SHP trials, we set the wrist at 45° pronation to allow natural grasp posture. The small object used in this task required a higher precision in reach-to-grasp in order to place the thumb accurately on the grasp surface. Similar to the large object, the object weight can be adjusted by inserting weight into the base of the object. Two object weights were used: Light (220 g) and Medium (420 g). The solid crushing threshold was again 80 N. The fragile crushing thresholds for the Light and Medium objects were 4 and 6 N, respectively. Subjects received same instruction as the large object pick and place regarding task objective. The kinematics of the object was tracked by motion capture system.

##### Compliant Object Squeeze

Subjects were instructed to repetitively squeeze a compliant object (Figure 2D) with power grasp. The object consisted of two curved grasp surfaces, which were connected by a pair of linear sliders and a spring. Therefore, the object only allows one dimensional deformation with maximum width of 8.5 cm (determined by a mechanical stop). The compliance of the object was determined by the stiffness of the spring, and two types of compliance were selected: Soft (0.33 N/mm) and Hard (0.54 N/mm). Visual feedback about the deformation of the object was given to the subjects on the monitor by tracking the positions of the grasp surfaces with motion capture system. To prepare for a trial, subjects had to lightly grasp the object (<0.2 cm deformation) with either their normal right hand or the SHP. For SHP trials, the wrist was set at 60° supination to allow natural grasp posture. On a “Go” signal, subjects were asked to match the target deformation shown on the monitor repetitively. There were two levels of target deformation 0.8 cm, and 1.8 cm with an error margin of 0.2 cm, each was presented five times within a trial. These two target levels alternated, and each level had to be maintained for 1 s continuously to automatically proceed to the next one.

### Experiment Procedure

Both SG and HG groups followed the exact same experimental procedure, and the only difference between the two groups was the myoelectric controllers. In experiment preparation, we placed the sEMG electrodes over the muscle bellies of the target muscles (i.e., FDS and EDC). The skin was cleaned with alcohol pads and the electrodes were secured by elastic medical tape. A calibration procedure was implemented by asking subjects to perform maximal voluntary isometric contraction (MVC) of the FDS or EDC. The onboard gains of the electrodes were adjusted such that the maximum output voltage represents the MVC of the corresponding muscle. In the baseline session, subjects performed all experimental tasks with their normal right hand wearing the same glove as the SHP glove, such that the friction conditions are matched. There were four conditions for Large object pick-and-place task: Heavy-Solid, Heavy-Fragile, Medium-Solid, and Medium-Fragile. Similarly, there were four conditions for Small object pick-and-place task: Medium-Solid, Medium-Fragile, Light-Solid, and Light-Fragile. Finally, there were two conditions for the Compliant object squeeze task: Soft and Hard. One baseline trial was performed for each of these ten conditions, with 1 min break given between conditions (see Table 2 for summary of conditions). The order of these conditions was randomized within each task for each subject. Most importantly, before each condition involving fragile object, subjects were given 15 s to understand the corresponding crushing threshold. Subjects were instructed to slowly ramping up the grasp force multiple times without lifting the object, until they heard the glass breaking sound. We also told subjects to memorize the fragility in association with the object type (e.g., Large Heavy) for the SHP trials later. For all baseline trials, we also recorded the position of the wrist center with the motion capture system, in addition to object positions, grasp forces, and sEMG.

**Table 2:** Summary of experimental conditions for both controller groups.

Native hand	Large object	Heavy (820 g)	Solid (80 N)	SoftHand-Pro	Large object	Heavy (820 g)	Solid (80 N)
			Fragile (9 N)				Fragile (9 N)

		Medium (420 g)	Solid (80 N)			Medium (420 g)	Solid (80 N)
			Fragile (6 N)				Fragile (6 N)

	Small object	Medium (420 g)	Solid (80 N)		Small object	Medium (420 g)	Solid (80 N)
			Fragile (6 N)				Fragile (6 N)

		Light (220 g)	Solid (80 N)			Light (220 g)	Solid (80 N)
			Fragile (4 N)				Fragile (4 N)

	Compliant object	Soft (0.33 N/mm)			Compliant object	Soft (0.33 N/mm)	
		Hard (0.54 N/mm)				Hard (0.54 N/mm)	

*Actual order was randomized*.

Following baseline session, subjects were fully equipped with the prosthetic system and went through two training tasks. One-minute break was given after each training task. After training session, subjects performed all ten experimental conditions again with SHP, with three consecutive trials per each condition (Table 2). The order of these conditions was also randomized within each task for each subject. In contrast to the baseline session, subjects were not allowed to explore the crushing threshold for the fragile objects, but only rely on their previous experience with the same object instead. For all baseline trials, we recorded the position of the wrist center of the SHP, object positions, grasp forces, SHP residual current, SHP motor position, CUFF motor current, and sEMG. All tasks were implemented using customized Matlab, C++, and LabView programs.

### Data Processing and Analysis

#### Experimental Variables

##### Training Tasks

For motion control training, we assessed the performance by computing the averaged time to perform each target action, which was defined as the time between the onsets of two consecutive target positions. Within each trial, there were four types of required change of motor positions (30°, 60°, 90°, and 120°) combined with two actions (open or close). Each specific action (e.g., open 60°) occurred twice, and we used the average time of the two as within trial performance. Note that the two controller groups had the same EMG-to-motion gains in this task, because the SHP is always in Free Motion state. For force control training, we assessed the performance by computing the total time to complete each trial. Additionally, we computed the averaged EMG magnitude as an indicator of motor effort within each trial.

#### Experimental Tasks

**Figure 3:**
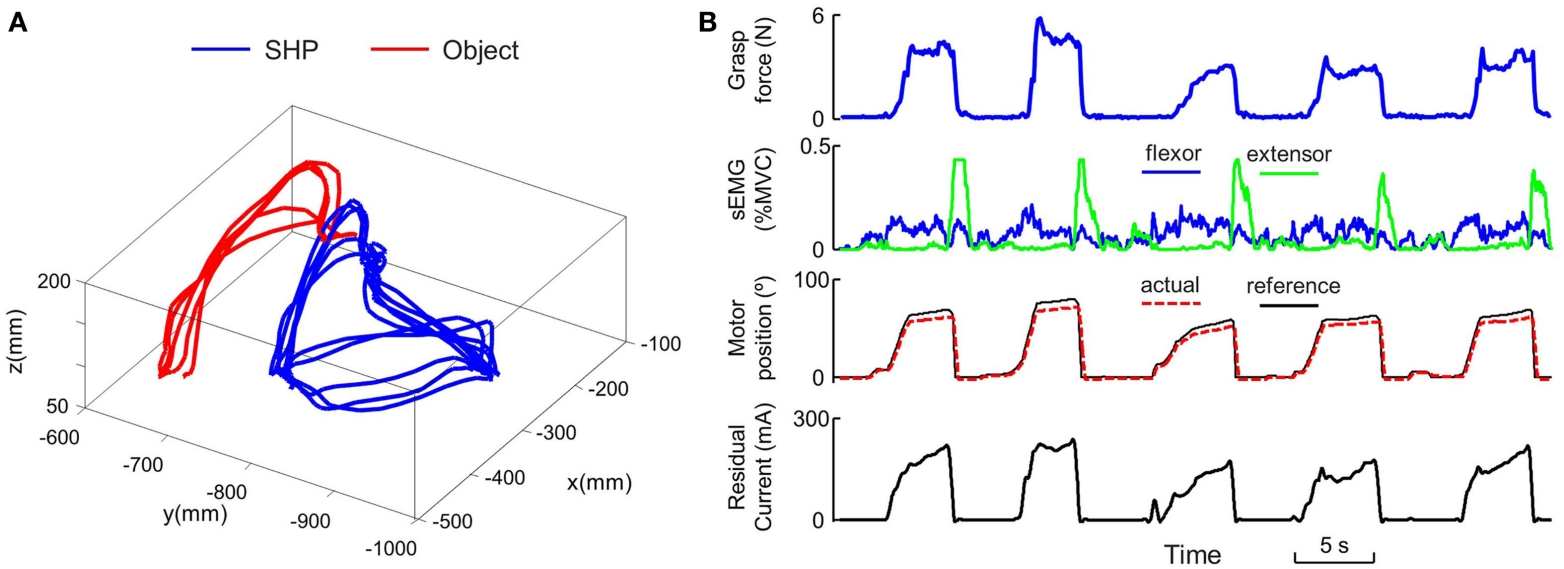
Sample experimental recording. **(A)** Representative 3-dimensional trajectory profile during object pick-and-place task. **(B)** Representative temporal profile of multiple experimental variables from one object pick-and-place trial.

For both object pick-and-place tasks, we mainly focus on the following measures. First, we use the number of successful transport completed within 45 s as the gross outcome measure. This is computed from both object marker data and object force sensor data, since successful completion requires no dropping (kinematics) or crushing (force) of the object between two target regions. Second, we assess hand-arm coordination by defining transport speed during successful transport. This is computed as wrist velocity at the time when the object is moving across the obstacle. Third, we assess the force modulation by defining grasp force during successful transport. This is computed as the force normal to the grasp surface at the time when the object is moving across the obstacle. Finally, we evaluate the myoelectric control by defining flexor activation, extensor activation, and co-contraction. These are computed as the average magnitude of the corresponding sEMG signals. Note that for SHP trials, each experimental condition consists of three trials and we take the average for these measures. A representative trial (sub13, Large object, Medium weight, Fragile) is shown in Figure 3. For the object squeeze task, we computed the time to complete one trial (i.e., five squeezes), as well as the averaged EMG magnitude for flexor, extensor, and co-contraction. A summary of variables is given in Table 3.

**Table 3:** Summary of experimental variables for both controller groups.

Motion training	Averaged time to perform each target open/close action
Force training	Total time to complete each trial
	Averaged EMG magnitude

Object pick and place	Number of successful transport completed within 45 s
	Grasp force during successful transport
	Transport speed during successful transport
	EMG activation of flexor
	EMG activation of extensor
	EMG activation of co-contraction

Object squeeze	Averaged time to complete each trial
	EMG activation of flexor
	EMG activation of extensor
	EMG activation of co-contraction

#### Statistical Analysis

One subject in the differential control group was excluded from the data analysis because he was not able to finish training within an acceptable performance range, therefore did not participate the experimental task with SHP. To compare between two controllers, we used mixed ANOVA with Group as the between subject factor and task conditions as within subject factors. We also used repeated measure ANOVA to assess benchmark performance with subject’s normal hand. *Post hoc* comparisons were used with Bonferroni correction when needed.

## Results

### Motion Control Training

**Figure 4:**
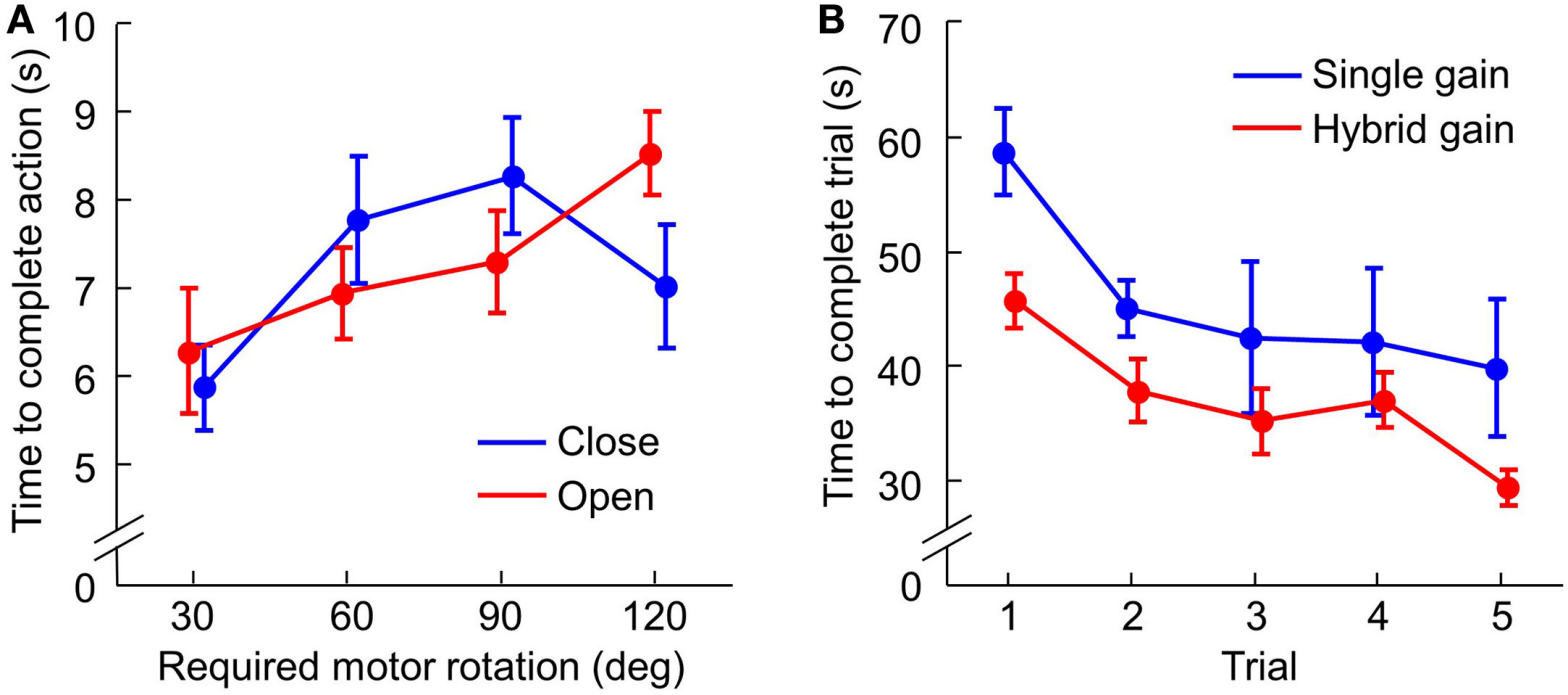
Motion and force training results. **(A)** The time to complete open and close of the SoftHand-Pro in motion training across four levels of required absolute change of the motor position, averaged across three trials. **(B)** The time to complete all force targets with two controllers across five training trials (mean ± SE).

The motion training was designed to familiarize subjects with the myoelectric controller. For both “open” and “close” actions, subjects were able to perform quite well from the beginning and we did not observe improvement over three training trials. With separate three-way mixed ANOVA (Group, Trial, and Target), we found only a significant effect of Target for both “close” and “open” actions (*p* = 0.001 and *p* < 0.001, respectively). This is expected, since both SG and HG groups used the same free motion controller that has been previously shown to be intuitive and efficient (Fani et al., 2016). Furthermore, we performed another three-way mixed ANOVA after averaging across trials (Group, Target, and Action). We found a significant Target × Action interaction (*p* = 0.015, Figure 4A). *Post hoc* T-test showed that 30° close took significantly shorter time than the other three closing actions, whereas 120° open took significantly longer than the other three opening actions (*p* < 0.05). This indicates that subjects were able to take advantages of the proportional control implemented for the SHP to scale the movement speed of the fingers as a function of the distance to be covered. Note that such scaling is an important feature observed in human when grasping object with different sizes (Bootsma et al., 1994).

### Force Control Training

Force training was designed to precisely generate desired grasp force with the help of visual feedback. Additionally, subjects could associate the haptic feedback from the CUFF to their own actions. Unlike motion control, force control with SHP was challenging in the beginning for both controller groups. The performance gradually improved over five training trials. Importantly, SG group performed consistently worse than the HG group (Figure 4B). These findings were confirmed by two-way ANOVA (Group and Trial) which showed significant effect of both Trial (*p* < 0.001) and Group (*p* = 0.048). Furthermore, we examined the average EMG used in force control training with two-way mixed ANOVA (Group and Trial). For both flexor and extensor muscles, we found HG group used significant larger activity than the SG group across training trials (main effect of Group *p* < 0.001 and *p* = 0.01; no effect of Trial). This result suggests that the hybrid controller allows better control of grasping force but requires greater effort/energy. We want to point out that, unlike natural grasping, here the energy is spent in modulating grasp force, but not maintaining grasp force, due to the nature of velocity-based myoelectric control.

### Object Pick-and-Place Tasks: Performance

**Figure 5:**
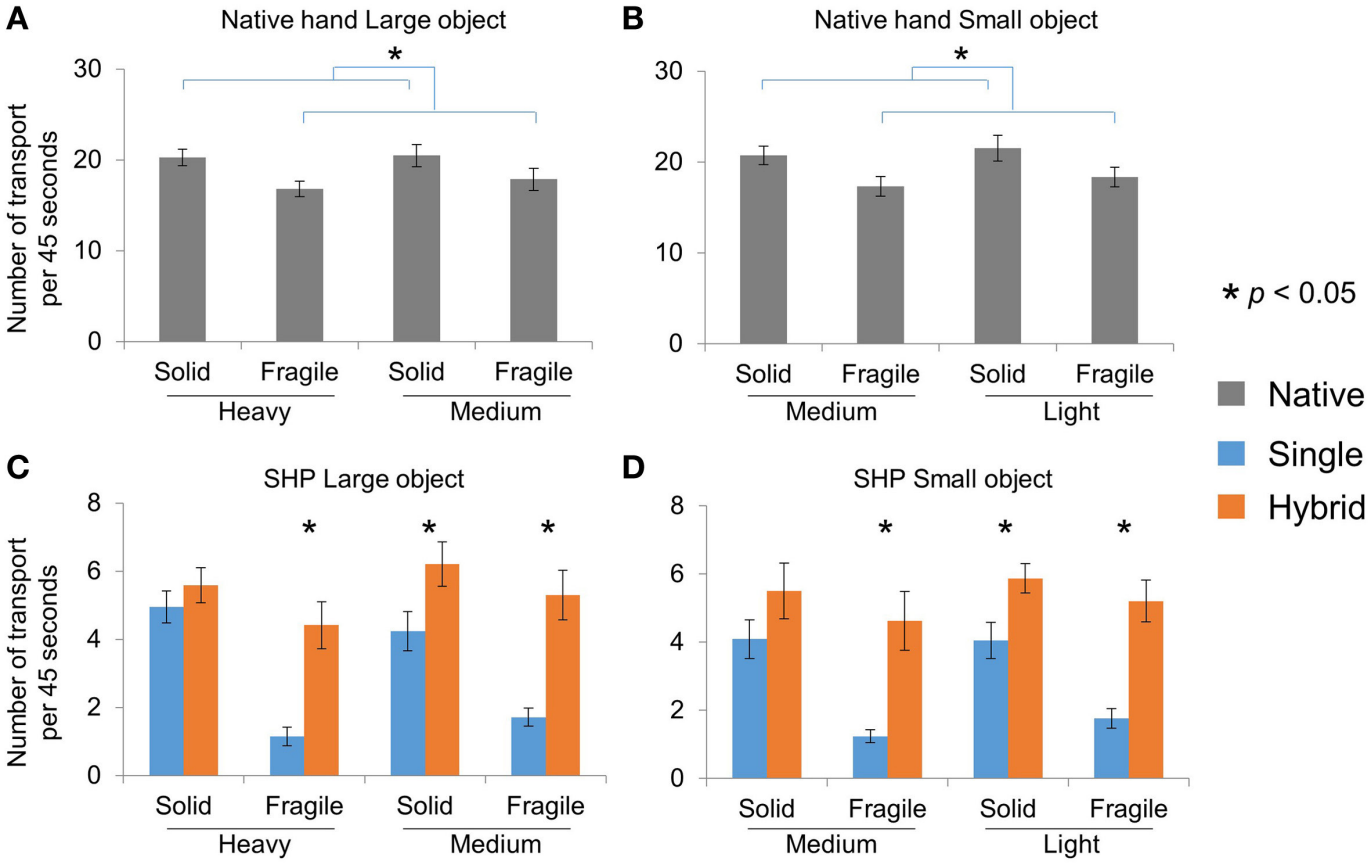
Number of successful transport in pick-and-place tasks. **(A)** and **(B)** Native hand performance for Large and Small objects, respectively. **(C)** and **(D)** SHP performance for Large and Small objects, respectively.

We first quantified subjects’ performance with their native hand. This allows us to establish benchmark behavior for our novel tasks, which is then used evaluate the SHP controllers. Similar benchmark quantifications will also be used in the following sections regarding different aspects of the object pick-and-place tasks. The overall task performance is assessed by the number of successful transport within 45 s, using three-way mixed ANOVA (Group, Weight, and Fragility) per object size. For both Large and Small object pick-and-place tasks, two groups performed equally well. Furthermore, only Fragility but not Weight of the objects played a role in the net performance (Figures 5A,B). We found that the number of successful transport for fragile objects is significantly less than the solid ones (only main effect of Fragility with both Large and Small object *p* < 0.001). This could be because subjects handled the fragile objects with more caution, thus being slower.

With SHP, both groups performed the tasks much slower than their native hands, and we found that HG controller outperformed SG controller when transporting fragile objects (Figures 5C,D). Specifically, with the Large object, we found significant Fragility × Group (*p* = 0.003) and Fragility × Weight interactions (*p* = 0.023). *Post hoc* comparisons suggested that the HG group performed significantly better than the SG group in Heavy-Fragile, Medium-Solid, and Medium-Fragile conditions (*p* < 0.05; Figure 5C). No difference was found between two groups in the Heavy-Solid condition. Similarly with the Small object, we found a significant Fragility × Group interaction (*p* = 0.035). Further *t*-test suggested that hybrid group performed significantly better than differential group in Heavy-Fragile, Medium-Solid, and Medium-Fragile conditions (*p* < 0.05; Figure 5D), but not in Heavy-Solid condition. Interestingly, we demonstrated a qualitatively similar pattern of Fragility effect between SHP and native hand in the HG group but not the SG group, despite of significantly less number of completion overall.

**Figure 6:**
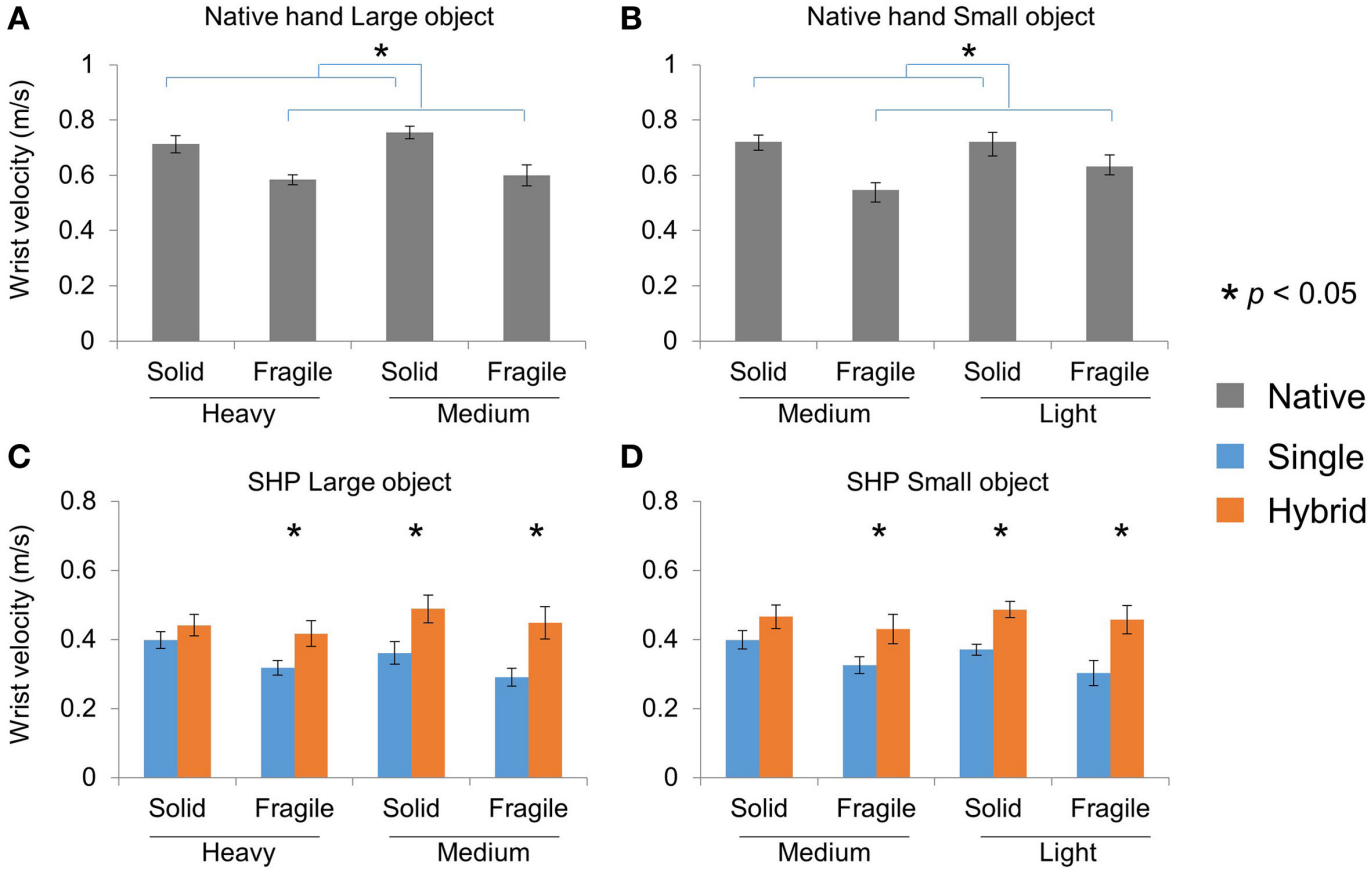
Wrist velocity in pick-and-place tasks. **(A)** and **(B)** Native hand wrist velocity for successful transport of Large and Small objects, respectively. **(C)** and **(D)** SHP wrist velocity for successful transport of Large and Small objects, respectively.

To further understand the difference between the HG and SG controller, we examined the hand-arm coordination using the velocity of the wrist center when the object was moving over the obstacle during successfully completed object transport. For the native hands, the velocity is significantly lower for Fragile objects than Solid objects (Figures 6A,B). Three-way mixed ANOVA (Group, Weight, and Fragility) showed only main effect of Fragility with both Large and Small objects (*p* < 0.001). With SHP, subjects were also moving slower with Fragile objects (main effect of Fragility, *p* = 0.003 and *p* = 0.005 for Large and Small objects, respectively). There were also significant Weight × Group interaction (*p* = 0.033 and *p* = 0.019 for Large and Small objects, respectively). *Post hoc* analyses showed that HG group was moving significantly faster in Heavy-Fragile, Medium-Solid, and Medium-Fragile conditions (*p* < 0.05, Figures 6C,D). These results suggest that the superior performance of HG controller can be partially attributed to the faster arm movement when holding an object.

### Object Pick-and-Place Tasks: Grasp Forces

**Figure 7:**
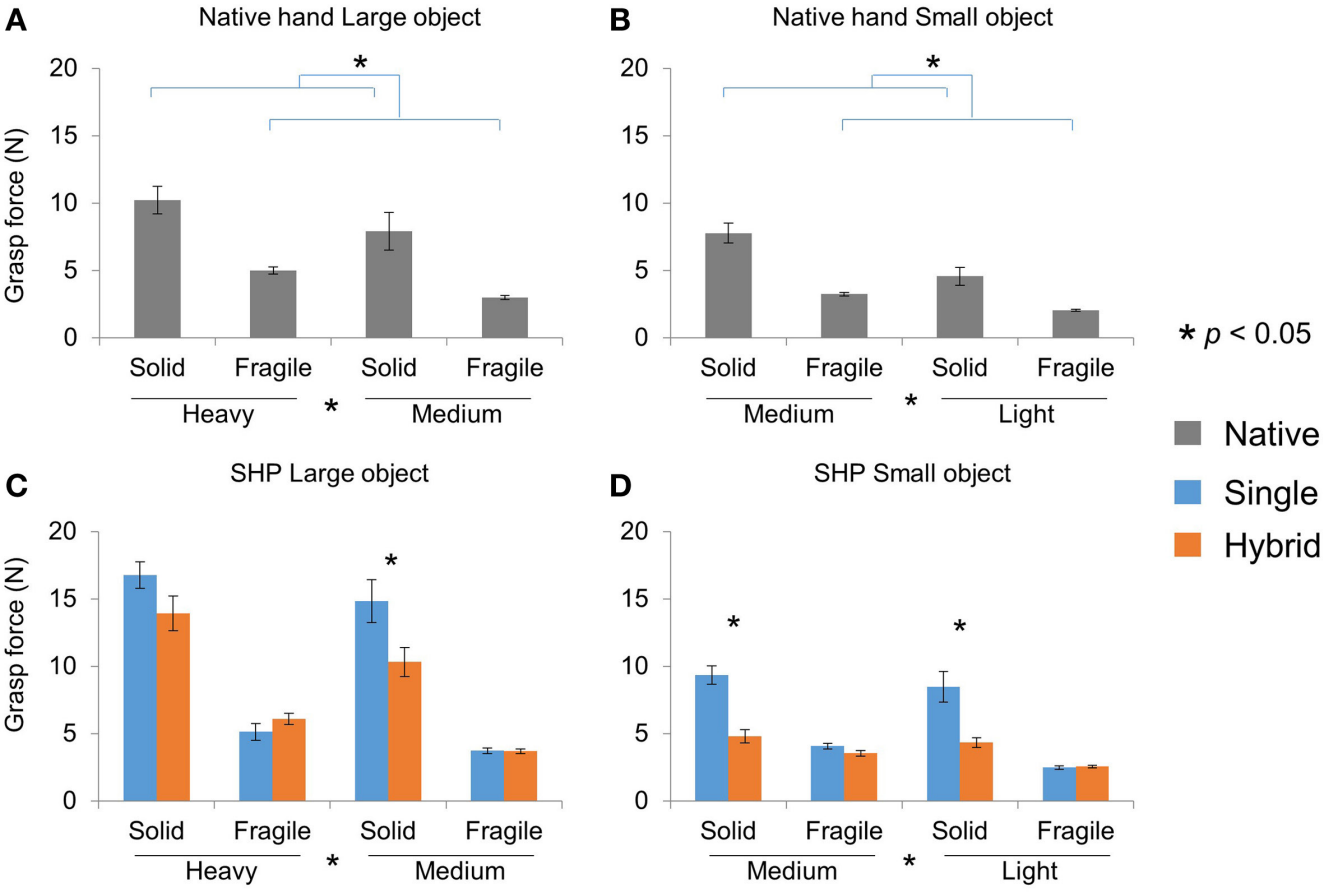
Grasp force in pick-and-place tasks. **(A)** and **(B)** Native hand grasp force for successful transport of Large and Small objects, respectively. **(C)** and **(D)** SHP grasp force for successful transport of Large and Small objects, respectively.

In addition to performance, we also measured grasp force when the object was moved over the obstacle during successfully completed object transport. For the native hands, we found that subjects scaled grasp force to object weight and fragility in both object size conditions (Figures 7A,B). Specifically, subjects used larger grasp force for heavier objects, and smaller grasp force when the object was fragile. These observations were confirmed by three-way mixed ANOVA (Group, Weight, and Fragility). With the Large object, there was a significant main effect of both Weight (*p* = 0.003) and Fragility (*p* < 0.001), but not Group. Similarly with the Small object, we found significant main effect of both Weight (*p* < 0.001) and Fragility (*p* < 0.001), but not Group.

With SHP, subjects were able to modulate grasp force in successful transport (Figures 7C,D). With the Large object, we found a main effect of Weight (*p* < 0.001), and a significant Fragility × Group interaction (*p* = 0.024). *Post hoc* comparisons showed that HG group used significantly smaller grasp force than the SG group in Medium-Solid condition (*p* < 0.05; Figure 4C). Similarly with the Small object, we also found a main effect of Weight (*p* = 0.003), and significant Fragility × Group interaction (*p* < 0.001). *t*-Test showed that HG group used significantly smaller grasp force than the SG group in both Medium-Solid and Light-Solid conditions (*p* < 0.05; Figure 4D). When compared with native hand, we found that HG group showed qualitative similar pattern of grasp force modulation, but SG group did not.

### Object Pick-and-Place Tasks: EMG

To better understand how subjects use their muscle activities with SG and HG controllers, we also compared the average EMG used in these tasks. Note that we do not use EMG from native hand here as benchmark because (1) velocity-based myoelectric control is different from natural muscle control by nature and (2) the two sEMG channels cannot provide comprehensive measure of the muscle activity from native hand used in these tasks (e.g., missing intrinsic muscles).

**Figure 8:**
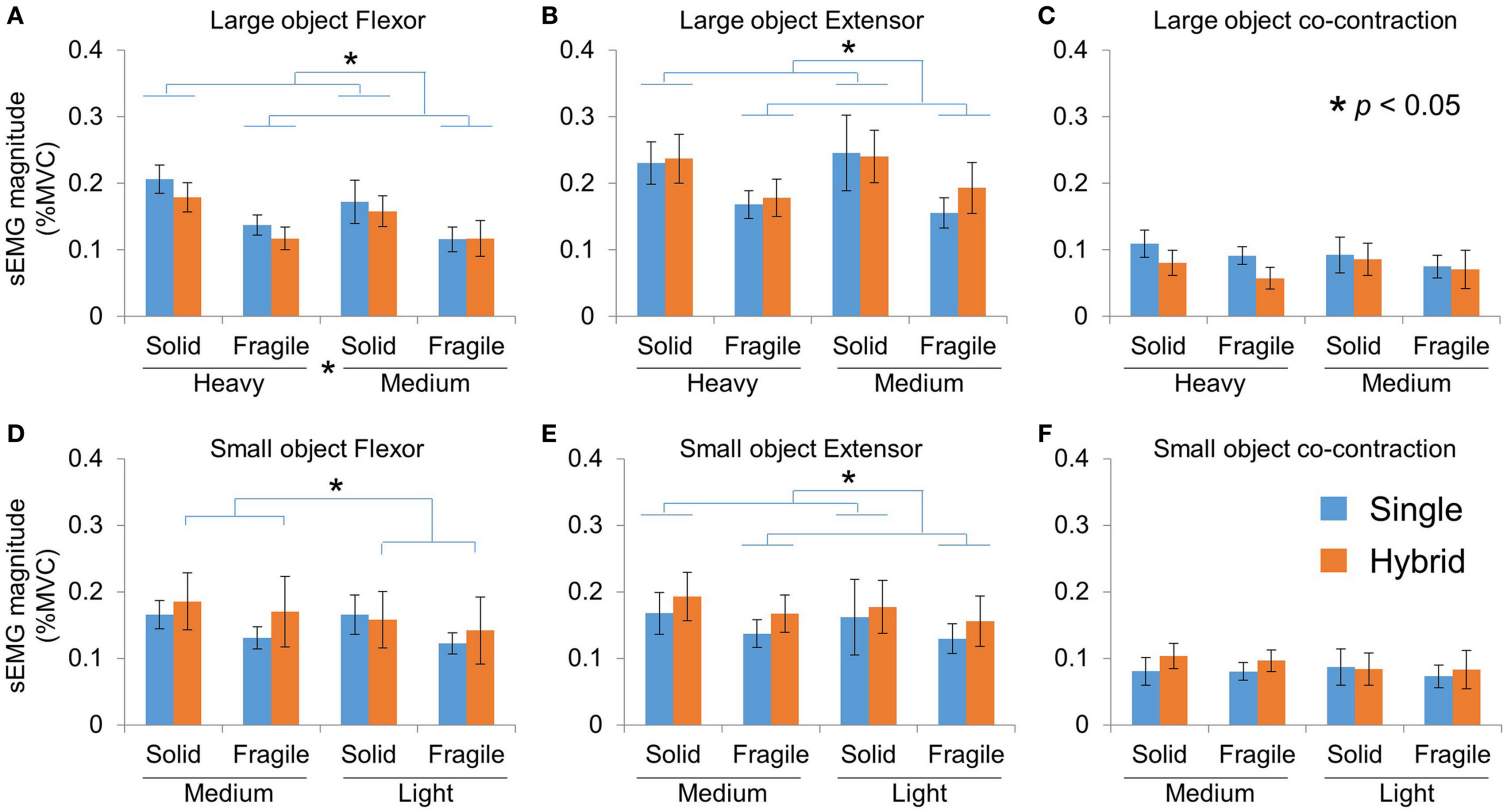
sEMG magnitude in pick-and-place tasks. **(A)**, **(B)**, and **(C)** sEMG activities of flexor, extensor, and co-contraction in Large object tasks, respectively. (**C)**, **(D)**, and **(E)** sEMG activities of flexor, extensor, and co-contraction in Small object tasks, respectively.

With the Large object, we found no difference in the average flexor EMG magnitude between the two controller groups (Figure 8A; three-way mixed ANOVA, only main effect of Weight and Fragility, *p* = 0.035 and *p* < 0.001, respectively). Furthermore, we found no difference in the extensor EMG magnitude between the two groups (Figure 8B; only main effect of Fragility *p* = 0.002). Finally, we found no difference in the co-contraction of the muscles between two groups (Figure 8C). With the Small object, we found a main effect of Fragility (*p* = 0.009) for the wrist flexor muscle, as well as a Group × Weight interaction (*p* = 0.038). *Post hoc* comparisons showed that subjects in the HG group used less EMG for light weight than for the medium weight, but the SG group did not show difference between weights (Figure 8D). For the wrist extensor muscle, we found no difference between the two controller groups (Figure 8E; only main effect of Fragility *p* = 0.005). Finally, we again found no difference in the co-contraction of the muscles between two groups (Figure 8F). To summarize, subjects used less EMG from both flexor and extensor muscles for fragile objects regardless of group (Figure 8#). This is expected because fragile objects require much smaller grasp force (Figures 8C,D), therefore less EMG was needed to drive the reference motor position. Interestingly, it was found that the flexor activity was scaled to object weight in all conditions for the HG group, but not SG group.

### Compliant Object Squeeze Task

**Figure 9:**
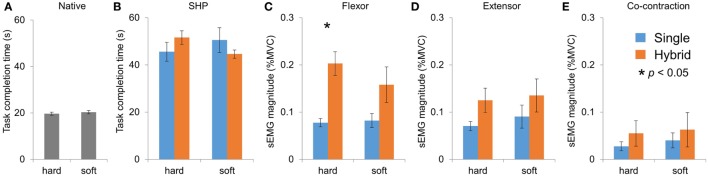
Performance and sEMG in compliant object squeeze tasks. **(A)** and **(B)** Completion times for Native hand and SHP, respectively. **(C)**, **(D)**, and **(E)** sEMG activities of flexor, extensor, and co-contraction in compliant object tasks, respectively.

In addition to object pick-and-place tasks, subject performed compliant object task in which they had to deform a compliant object with either their native hand or the SHP. There were two levels of compliance that were set by the stiffness of the spring inside the object (soft and hard, 0.33 and 0.54 N/mm, respectively). There was no difference between the two compliance levels for the native hand (Figure 9A). For the SHP, we found that single gain and the HG controller performed similarly in this task, and both were much slower than their native hands (Figure 9B). Two-way mixed ANOVA (Group and Compliance) showed a significant Group × Compliance interaction (*p* = 0.032). However, *post hoc* comparison between two compliance levels did not reveal significant differences.

We also compared the average EMG between the two controller groups with two-way mixed ANOVA (Group and Compliance). For the flexor muscle, we found that subjects used significant larger activity in the hybrid controller group (Figure 9C; only main effect of Group, *p* = 0.011). For the extensor activity and co-contraction, no significant difference was found between the two groups (Figures 9D,E).

## Discussion

The goal of this study was to improve the myoelectric control of grasp forces in functional tasks using a soft synergy-based prosthetic hand. Specifically, we developed and implemented a HG controller and compared its performance to a previously validated conventional SG controller. We demonstrated that the new controller (a) significantly improved subjects’ ability to perform fine force control when transporting objects with different shapes, weights and fragility (Figure 5) and (b) qualitatively demonstrated natural modulation of grasp force in response to object’ physical property, such as weight (Figure 7). We discuss our results and future work below.

### Quantitative Assessment of Performance of Hand Prosthesis with Functional Tasks

To meet the needs of creating reliable, functional, and robust hand prostheses, it is important to assess their performance in functional tasks that require physical interaction with objects, as well as coordination of arm and hand. This is because the use of prosthetic hand in ADL often involves dynamic and unstructured environments, in which the effect of gravity and object physical properties needs to be properly compensated. There are several clinical assessment tools available, such as Southampton Hand Assessment Procedure, Box and Block Test, and Jebsen Hand Function Test. However, these tests are usually scored based on gross measures, such as task completion time or quality of movement. As such, they do not provide information about how the tests are completed (i.e., movement kinematics, grasp force). Additionally, these tasks typically do not assess subjects’ ability to control grasp force, which plays an important role in ADL. Researchers have recently started to incorporate motion capture and force sensors to quantify and standardize the evaluation of the hand–object interactions during use of prosthetic hands (Hebert and Lewicke, 2012; Engeberg and Meek, 2013; Fani et al., 2016; Godfrey et al., 2016; Wilson et al., 2017). Such quantitative assessment can identify potential bottlenecks and issues within the complex integration among hardware, control, and human user input, therefore helping to validate and optimize the prosthetic systems. In the current study, we developed a set of novel functional tasks that aimed to quantify the capability of prosthetic system to control grasp forces. The advantages of our tasks are threefolds. First, our tasks use objects that can be easily adjusted to cover a wide range of different physical properties, such as size, weight, and fragility. This allows us to test the versatility of the function of a prosthetic hand. Second, our tasks require repetitive dynamic actions similar to the Box and Block Test, which can be used to assess the reliability and robustness of the prosthesis. Third, our setup is fully equipped with both motion capture and force sensing technologies, thus being able to capture multiple dimensions of the task performance. Furthermore, our experimental design also allows comparison between the prosthetic system and benchmark performance from the native hands. We believe that the ability of a prosthetic system to exhibit human-like kinematic and kinetic behavior is critical for the acceptance of the terminal device.

### Improved Force Control with Context-Dependent HG Controller

Fine grasp force control is a defining feature in human’s manual dexterity. When grasping and moving an object, it is well known that the grasp force is regulated to the object’s weight and friction. Specifically, there is a minimum level of grasp force required to prevent object slip, given a weight and friction coefficient combination. The applied grasp force is normally slightly higher than the minimally required, demonstrating a consistent “safety margin” which balances energy efficiency and slip prevention (Johansson and Westling, 1984; Westling and Johansson, 1984). When friction is constant as in our study, such grasp force control will lead to the natural scaling of grasp force to object weight (e.g., larger grasp force on heavier objects). Indeed, in current study we showed that subjects were able to modulate grasp force in response to object weight with their native hands even when wearing a glove (Figure 7). Extensive investigation has revealed that weight specific grasp force control is achieved by a combination of memory based feed-forward control and sensory feedback driven corrections. During initial encounter with a novel object, feedforward motor command can be generated based on the object’s physical properties which are visually estimated using previous experiences with similar objects (Gordon et al., 1993). If the motor command is erroneously programmed due to inaccurate estimation, the central nervous system (CNS) can use somatosensory feedback to generate corrective responses after contact and/or after lift (Johansson and Westling, 1988; Johansson and Cole, 1992). After repetitive interaction with the same object, internal representation of the object properties can be formed and used to generate more precise feedforward motor command in the following interactions (Flanagan et al., 2001). Importantly, the “safety margin” for grasp force can also be flexibly adjusted in a feedforward fashion to account for uncertainty in the dynamic environment (Hadjiosif and Smith, 2015), or the fragility of objects (Gorniak et al., 2010). In the current study, subjects used much less grasp force on the fragile object than on the solid ones (assuming same object weight) with their native hands. Such drop of “safety margin” was accompanied by decreased arm movement speed, which is consistent with previous findings (Gorniak et al., 2010).

There are two common ways to enable grasp force control in prosthetic hands. The first approach is fully automated by the implementation of force feedback loop using force and position sensors (Engeberg et al., 2008, 2009; Engeberg and Meek, 2013). While the accuracy and reliability of automated force control is very good in single degrees of freedom rigid prosthetic hand (e.g., Motion Control Hand), it is challenging to scale this approach up to multi-finger hands and/or hands with embedded compliance (e.g., SHP) due to complex hand–object interactions. Alternatively, the force control can be fully operated by the user with some form of haptic feedback about the grasp force [for review, see Antfolk et al. (2013) and Li et al. (2017)], such as vibrotactile stimulation (Rombokas et al., 2013; Lum et al., 2014), electrotactile stimulation (Wang et al., 1995), mechanotactile stimulation (Ajoudani et al., 2014; Casini et al., 2015), and direct nerve stimulation (Raspopovic et al., 2014). In most cases, the feedback signal carries continuous information about grasp force, allowing subjects to reduce grasp force or perceive object softness. A more recent study also showed that discrete feedback about mechanical events during hand-object interaction is sufficient to allow user to better handle fragile object (Clemente et al., 2015). However, most of the studies used relatively static and/or constrained tasks, and have not tested the user’s ability to integrate haptic feedback in force control during highly dynamic tasks across a range of different object size and weight. In the current study, we showed that our novel hybrid controller, paired with a soft synergy-based hand and continuous mechanotactile feedback can achieve this goal. Most importantly, to the best of our knowledge, we are the first to demonstrate human-like grasp force modulation to object weight.

Instead of tuning the haptic feedback, our approach focused on the design of EMG-to-motor control interface. This is because we acknowledge the importance to enable users to (1) accurately generate anticipatory motor command and (2) to make fine corrective motor response after receiving sensory feedback. Both of which are crucial to human’s manual dexterity, as reviewed in the beginning of this section. Furthermore, we propose that, in a proportional EMG control scheme as in SHP, the EMG-to-motor control mapping has to be optimized separately for free motion and grasp force due to distinct behavior of the motor during motion and force generation. Note that the modulation of EMG-to-motor mapping can be designed to fully rely on the user, such as the concept of “teleimpednace” where muscle co-contraction is used to change the mapping (Ajoudani et al., 2014). However, this would increase the complexity of the myoelectric interface, leading to higher demand in attention to simultaneously control multiple variables. It has been shown that a trade-off has to be made when deciding the level of sharing of control between the user and the hardware, and an intermediate level of interaction between the two was favored (Cipriani et al., 2008). Following this idea, context-dependent switching scheme can be found in several recent studies to control kinematics of the prosthetic hands based on sEMG pattern (Amsuess et al., 2016), limb kinematics and/or grasp force (Jiang et al., 2013; Patel et al., 2017), or vision (Markovic et al., 2014, 2015; Ghazaei et al., 2017). It has been argued that such semi-autonomous shared control can help to shield some low level execution details and decreases cognitive burden while maintaining high level function (Castellini et al., 2014). We agree with this assessment, and furthermore believe that the prosthetic system needs to merge both sensory and motor information to best determine the context of operation, including both sensors in the hardware and the sEMG from user input. Therefore, we choose to improve prosthesis force control by implementing a “context aware” controller that changes the EMG-to-motor mapping based on both the condition of the hand (i.e., free motion or object grasping) and the intent of the user (i.e., open or close). We note that the state switching rules were relatively simple in our study due to limited sensing capability associated with the design goal of enabling intuitive control of the SHP Nevertheless, the present work provides proof-of-concept evidence that human-like force control can be achieved using the proposed approach.

### Effort-Performance Trade-Off in Myoelectric Control of Hand Prosthesis

We want to point out that the superior performance of the hybrid controller came at a cost of increased demand of energy (i.e., muscle activation at higher amplitude and for longer time). However, this is not necessarily undesired. In fact, our result where subjects scale grasp force to object weight indicates that the increased energy demand from our controller effectively evoke the CNS’s ability to optimize the motor command for energy efficiency. This indirectly leads to lower energy consumption in the prosthetic hand as the grasp force is subsequently optimized, which can extend the usage time thanks to less battery consumption and reduced tension of the driving tendon.

### Conclusion and Future Work

Our results provide strong support to the functional advantage of a context-dependent myoelectric interface for the control of grasp force during hand–object interactions. Although the controller was only tested with a soft-synergy based prosthetic hand and a mechanotactile feedback system, we believe that it can be extended to other terminal devices and feedback systems, including next generation of SHP with multiple actuators (Delia Santina et al., 2015) and direct nerve stimulation. Future work includes, but not limited to, finding optimal EMG-to-motor mapping parameters in different sensorimotor states, better state definition and transitions, as well as determining the level of control sharing between user and hardware.

## Ethics Statement

This study was carried out in accordance with the recommendations of “Institutional Review Board at Arizona State University” with written informed consent from all subjects. All subjects gave written informed consent in accordance with the Declaration of Helsinki. The protocol was approved by the “Institutional Review Board at Arizona State University.”

## Author Contributions

QF and MS designed the study and wrote the manuscript. QF implemented the controllers of the SoftHand Pro, built the experimental setup, and performed the experiments and data analysis.

## Conflict of Interest Statement

The authors declare that the research was conducted in the absence of any commercial or financial relationships that could be construed as a potential conflict of interest.
